# Predictors of uveitic macular edema and functional prognostic outcomes: real-life data from the international AIDA Network uveitis registry

**DOI:** 10.3389/fmed.2025.1609613

**Published:** 2025-07-30

**Authors:** Jurgen Sota, Germán Mejía-Salgado, Silvana Guerriero, Gaafar Ragab, Stefania Costi, Maria Pia Paroli, Andrea Hinojosa-Azaola, Giuseppe Lopalco, Luciana Breda, Henrique Ayres Mayrink Giardini, Alex Fonollosa, Maria Sole Chimenti, Antonio Vitale, Carla Gaggiano, Blanca Aguilar-Barrera, Laura Daniela Rodríguez-Camelo, Guillermo Arturo Guaracha-Basañez, Mohamed Tharwat Hegazy, Rosanna Dammacco, Valeria Albano, Eduardo Martín-Nares, Santiago Espinosa-Lugo, Mahmoud Ghanema, Maria Morrone, Saverio La Bella, Rafael Alves Cordeiro, Francesco Carubbi, Alessandro Conforti, Piero Ruscitti, Ibrahim AlMaglouth, Rosaria Talarico, Stefano Gentileschi, Petros P. Sfikakis, Valeria Caggiano, Matteo Piga, Adele Civino, Francesca Ricci, Maissa Thabet, Marcello Govoni, Abdurrahman Tufan, Francesca Crisafulli, Jessica Sbalchiero, Sulaiman M. Al-Mayouf, Angela Mauro, Soad Hashad, Francesca Minoia, Alma Nunzia Olivieri, Samar Tharwat, Maria Cristina Maggio, Abdelhfeez Moshrif, Gian Domenico Sebastiani, Daniela Opris-Belinski, Gülen Hatemi, Haner Direskeneli, Fatma Alibaz-Öner, Lampros Fotis, José Hernández-Rodríguez, Giovanni Conti, Piercarlo Sarzi Puttini, Ombretta Viapiana, Annarita Giardina, Patrizia Barone, Kalpana Babu, Rana Hussein Amin, Perla Ayumi Kawakami-Campos, Vishali Gupta, Annamaria Iagnocco, Ali Şahin, Antonella Insalaco, Andrés González-García, Ezgi Deniz Batu, Ester Carreño, Emanuela Del Giudice, Cecilia Beatrice Chighizola, Fabrizio Conti, Alberto Balistreri, Bruno Frediani, Luca Cantarini, Alejandra de-la-Torre, Claudia Fabiani

**Affiliations:** ^1^Rheumatology Unit, Department of Medical Sciences, Surgery, and Neurosciences, University of Siena and Azienda Ospedaliero-Universitaria Senese [European Reference Network (ERN) for Rare Immunodeficiency, Autoinflammatory, and Autoimmune Diseases (RITA) Center], Siena, Italy; ^2^Neuroscience Research Group (NEUROS), Neurovitae Center for Neuroscience, Institute of Translational Medicine (IMT), School of Medicine and Health Sciences, Universidad del Rosario, Bogotá, Colombia; ^3^Department of DiBrain, University of Bari, Bari, Italy; ^4^Unit of Rheumatology and Clinical Immunology, Department of Internal Medicine, Faculty of Medicine, Cairo University, Giza, Egypt; ^5^Faculty of Medicine, Newgiza University, Giza, Egypt; ^6^Unit of Pediatric Rheumatology, ASST Gaetano Pini-CTO, Milan, Italy; ^7^Uveitis Unit, Department of Sense Organs, Eye Clinic, Sapienza University of Rome, Rome, Italy; ^8^Department of Immunology and Rheumatology, Instituto Nacional de Ciencias Médicas y Nutrición Salvador Zubirán, Mexico City, Mexico; ^9^Department of Precision and Regenerative Medicine and Ionian Area (DiMePRe-J), Policlinic Hospital, University of Bari, Bari, Italy; ^10^Department of Paediatrics, University of Chieti-Pescara, Chieti, Italy; ^11^Division of Rheumatology, Faculdade de Medicina, Hospital das Clinicas (HCFMUSP), Universidade de São Paulo, São Paulo, Brazil; ^12^Department of Ophthalmology, Biocruces Bizkaia Health Research Institute, Cruces University Hospital, University of the Basque Country, Barakaldo, Spain; ^13^Rheumatology, Allergology, and Clinical Immunology, Department of Systems Medicine, University of Rome Tor Vergata, Rome, Italy; ^14^Department of Life, Health and Environmental Sciences, Division of Internal Medicine and Nephrology, ASL1 Avezzano-Sulmona-L'Aquila, San Salvatore Hospital, University of L'Aquila, L'Aquila, Italy; ^15^U.O. Medicina Generale, Ospedale San Paolo di Civitavecchia, Civitavecchia, Rome, Italy; ^16^Unit of Rheumatology, Department of Biotechnological and Applied Clinical Sciences, University of L'Aquila, L'Aquila, Italy; ^17^Unit of Rheumatology, Department of Medicine, King Saud University, Riyadh, Saudi Arabia; ^18^Unit of Rheumatology, Azienda Ospedaliero-Universitaria Pisana, Pisa, Italy; ^19^Joint Academic Rheumatology Program, National and Kapodistrian University of Athens Medical School [European Reference Network (ERN) for Rare Immunodeficiency, Autoinflammatory and Autoimmune Diseases (RITA) Center], Athens, Greece; ^20^Unit of Rheumatology, Department of Medical Sciences and Public Health, University and AOU of Cagliari, Cagliari, Italy; ^21^Unit of Pediatric Rheumatology and Immunology, Vito Fazzi Hospital, Lecce, Italy; ^22^Pediatric Clinic, University of Brescia and Spedali Civili di Brescia [European Reference Network (ERN) for Rare Immunodeficiency, Autoinflammatory, and Autoimmune Diseases (RITA) Center], Brescia, Italy; ^23^Department of Internal Medicine, Faculty of Medicine of Sousse, Farhat Hached University Hospital, University of Sousse, Sousse, Tunisia; ^24^Unit of Rheumatology, Department of Medical Sciences, Azienda Ospedaliero-Universitaria S. Anna-Ferrara, University of Ferrara, Ferrara, Italy; ^25^Division of Rheumatology, Department of Internal Medicine, Gazi University Hospital, Ankara, Türkiye; ^26^Rheumatology and Clinical Immunology, Spedali Civili and Department of Clinical and Experimental Sciences, University of Brescia [European Reference Network (ERN) for Rare Immunodeficiency, Autoinflammatory and Autoimmune Diseases (RITA) Center], Brescia, Italy; ^27^Department of Pediatrics, King Faisal Specialist Hospital and Research Center, College of Medicine, Alfaisal University, Riyadh, Saudi Arabia; ^28^Department of Biomedical and Clinical Sciences, Fatebenefratelli Hospital, Università di Milano, Milan, Italy; ^29^Unit of Pediatric Rheumatology, Department of Childhood and Developmental Medicine, Fatebenefratelli-Sacco Hospital, Milan, Italy; ^30^Department of Rheumatology, Tripoli Children Hospital, Tripoli, Libya; ^31^Department of Rheumatology, University of Tripoli, Tripoli, Libya; ^32^Fondazione IRCCS Ca' Granda Ospedale Maggiore Policlinico, Milan, Italy; ^33^Department of Woman, Child and of General and Specialized Surgery, University of Campania "Luigi Vanvitelli", Naples, Italy; ^34^Unit of Rheumatology and Immunology, Department of Internal Medicine, Mansoura University, Mansoura, Egypt; ^35^Department of Internal Medicine, Faculty of Medicine, Horus University, New Damietta, Egypt; ^36^University Department of Health Promotion, Mother and Child Care, Internal Medicine and Medical Specialties (PROMISE) "G. D'Alessandro", University of Palermo, Palermo, Italy; ^37^Department of Rheumatology, Faculty of Medicine, Al-Azhar University, Assiut, Egypt; ^38^U.O.C. Reumatologia, Ospedale San Camillo-Forlanini, Rome, Italy; ^39^Department of Rheumatology and Internal Medicine, Carol Davila University of Medicine and Pharmacy, Bucharest, Romania; ^40^Department of Internal Medicine, Division of Rheumatology, Cerrahpasa Medical School, Istanbul University-Cerrahpasa, Istanbul, Türkiye; ^41^Behçet's Disease Research Center, Istanbul University-Cerrahpasa, Istanbul, Türkiye; ^42^Department of Internal Medicine, Division of Rheumatology, School of Medicine, Marmara University, Istanbul, Türkiye; ^43^Department of Pediatrics, Attikon General Hospital, National and Kapodistrian University of Athens, Athens, Greece; ^44^Autoinflammatory Diseases Clinical Unit, Department of Autoimmune Diseases, Center of the European Reference Network (ERN) for Rare Immunodeficiency, Autoinflammatory and Autoimmune Diseases (RITA), Hospital Clinic of Barcelona, Institut d'Investigacions Biomèdiques August Pi i Sunyer (IDIBAPS), University of Barcelona, Barcelona, Spain; ^45^Unit of Pediatric Nephrology and Rheumatology, Azienda Ospedaliero Universitaria (AOU) G Martino, Messina, Italy; ^46^Unit of Rheumatology, Ospedale Sacco, Milan, Italy; ^47^Unit of Rheumatology, Department of Medicine, University and Azienda Ospedaliera Universitaria Integrata of Verona, Verona, Italy; ^48^UOC Medicina Interna, Ambulatorio di Reumatologia, ARNAS Civico Di Cristina Benfratelli, Palermo, Italy; ^49^Department of Clinical and Experimental Medicine, University of Catania, Catania, Italy; ^50^Department of Uveitis and Ocular Inflammation, Prabha Eye Clinic and Vittala International Institute of Ophthalmology, Bangalore, India; ^51^Department of Opthalmology, Faculty of Medicine, Cairo University, Giza, Egypt; ^52^Department of Ophthalmology, Instituto Nacional de Ciencias Médicas y Nutrición Salvador Zubirán, Mexico City, Mexico; ^53^Advanced Eye Centre, Postgraduate Institute of Medical Education and Research, Chandigarh, India; ^54^Academic Rheumatology Center, Dipartimento Scienze Cliniche e Biologiche, Università Degli Studi di Torino, Turin, Italy; ^55^Division of Rheumatology, Department of Internal Medicine, Sivas Cumhuriyet University Medical Faculty, Sivas, Türkiye; ^56^Division of Rheumatology, Ospedale Pediatrico Bambino Gesù, IRCCS [European Reference Network (ERN) for Rare Immunodeficiency, Autoinflammatory and Autoimmune Diseases (RITA) Center], Rome, Italy; ^57^Department of Internal Medicine, Autoimmune and Rare Diseases Unit, Hospital Universitario Ramón y Cajal, Universidad de Alcalá de Henares, IRYCIS, Madrid, Spain; ^58^Unit of Pediatric Rheumatology, Department of Pediatrics, Hacettepe University School of Medicine, Ankara, Türkiye; ^59^Department of Ophthalmology, Fundación Jiménez Díaz University Hospital (FJD), Madrid, Spain; ^60^Unit of Pediatric and Neonatology, Department of Maternal Infantile and Urological Sciences, University of Rome La Sapienza, Rome, Italy; ^61^Department of Clinical Sciences and Community Health, Research Center for Adult and Pediatric Rheumatic Diseases, University of Milan, Milan, Italy; ^62^Unit of Pediatric Rheumatology, Azienda Socio-Sanitaria Territoriale (ASST) Gaetano Pini Centro Specialistico Ortopedico Traumatologico (CTO), Milan, Italy; ^63^Unit of Rheumatology, Department of Clinical Internal, Anesthesiologic and Cardiovascular Sciences, Sapienza University of Rome, Rome, Italy; ^64^Bioengineering and Biomedical Data Science Lab, Department of Medical Biotechnologies, University of Siena [European Reference Network (ERN) for Rare Immunodeficiency, Autoinflammatory, and Autoimmune Diseases (RITA) Center], Siena, Italy; ^65^Unit of Ophthalmology, Department of Medicine, Surgery, and Neurosciences, University of Siena and Azienda Ospedaliero-Universitaria Senese [European Reference Network (ERN) for Rare Immunodeficiency, Autoinflammatory, and Autoimmune Diseases (RITA) Center], Siena, Italy

**Keywords:** uveitis, macular edema, visual acuity, registries, retinal vasculitis

## Abstract

**Objectives:**

To detect factors capable of predicting the development of macular edema (ME) throughout the disease course in patients affected by non-infectious uveitis (NIU).

**Methods:**

Predictive factors leading to the development of ME were analyzed through regression analysis. The functional impact of ME on best corrected visual acuity (BCVA) was also examined.

**Results:**

A total of 1,160 NIU patients (1,857 eyes) were analyzed. ME was observed in 148 (12.76%), affecting 211 eyes. It was significantly more frequent in patients with non-anterior NIU (*p* < 0.0001, RR = 4.01), retinal vasculitis (*p* < 0.0001), and other structural complications (*p* = 0.0005). Gender, HLA-B*27 and/or HLA-B*51 positivity, and ethnicity did not show any significant impact on the prevalence of ME (*p* = 0.635, *p* = 0.372, *p* = 0.193, respectively). Four variables were associated with ME development during NIU course: the non–anterior anatomical pattern (*p* < 0.0001, OR = 4.01), the presence of retinal vasculitis (*p* = 0.028, OR = 1.68), complications other than ME (*p* = 0.044, OR = 1.51) and immunosuppressive treatment (*p* = 0.010, OR 1.69) while the diagnosis of Behçet disease-related uveitis was less likely to be associated with ME development (*p* = 0.24, OR 0.545). Mean ± SD BCVA was significantly lower in eyes with ME (0.82 ± 0.30) compared to eyes without ME (0.71 ± 0.33).

**Conclusion:**

ME can develop across all NIU types, but is more likely in cases involving the posterior segment and retinal vasculitis. Regular and focused monitoring is recommended for these high-risk patients. The study also highlights the limited predictive value of demographic and HLA-related factors, helping refine clinical risk stratification and predictive modeling in NIU.

## Introduction

Macular edema (ME) consists of intra- or subretinal fluid accumulation in the macular region and represents a severe complication of many disorders affecting the retina, including uveitis. The pathophysiology of ME in uveitis is linked to the Starling equation and disruptions in the delicate balance, due to increased vascular leakage or decreased fluid resorption, which can lead to fluid accumulation, especially in the fovea, followed by the development of ME. Contributing factors to uveitic ME are both inflammatory and structural, including breakdown of the inner blood-retinal barrier, dysfunction of the retinal pigment epithelium (RPE), and the release of pro-inflammatory cytokines, which promote fluid and protein leakage into the extracellular space (1). The condition may result in progressive loss of macular integrity and, consequently, visual loss if treated improperly (1–3). ME emerges as a major cause of visual loss among the various structural complications arising during uveitis, accounting for 40% of visual impairment in patients affected by intraocular inflammation (1). Vision loss is potentially reversible with proper treatments, making timely detection and prompt intervention of paramount importance. However, in some instances, uveitic ME may persist or relapse despite optimal control of intraocular inflammation (4). In this context, identifying predictive factors for the ME development in patients affected by non-infectious uveitis (NIU) is crucial for an early intervention and a personalized treatment approach, to provide valuable insights in detecting high-risk patients prone to visual impairment and ideally anticipating its impact on visual prognosis. Despite the recognized relevance and importance, medical literature on this topic is limited. In the present study, we report our multicenter experience on the factors associated with ME and predictors of its development during NIU, based on a large cohort of patients enrolled in the international Autoinflammatory Disease Alliance (AIDA) registry for Behçet’s disease and in the international AIDA registry for uveitis. This large, real-world registry-based cohort study analyzes the longitudinal development of ME in NIU. It identifies and quantifies key clinical predictors while highlighting underreported non-significant associations and provides practical insights for daily clinical management.

## Materials and methods

### Study design, participants, and data collection

Medical records of patients affected by NIU, either idiopathic, associated with an immune-mediated systemic disease, or oculo-specific entities, were reviewed. Data, collected retrospectively and prospectively, were retrieved from the AIDA International Network Registries on ocular inflammatory disorders. More specifically, we extracted data collected up to August 18th, 2024, from the international AIDA Network for BD registry and for the Uveitis registry. These registries are clinical physician-driven and electronic-based instruments implemented for the retrospective and prospective collection of real-life data about demographics, socioeconomic status, clinical, laboratory, instrumental, and therapeutic information (5, 6). Uveitis was classified according to the Standardization of uveitis Nomenclature criteria (7). The following data were collected: gender, ethnic origin, age at onset, age at diagnosis, associated systemic disease, human leukocyte antigen (HLA)-typing, ocular complications developed over time from disease onset to the last follow-up visit, retinal vasculitis, best corrected visual acuity (BCVA) measured on Snellen charts and expressed in decimals, uveitis anatomical classification, number of relapses, treatments given throughout disease course. Medical charts with more than 20% of missing values were excluded from the study. Patients with diabetic retinopathy, hypertensive retinopathy, those receiving medications associated with the development of ME, or those who had undergone intraocular surgery or laser treatment within the past 3 months, potentially leading to ME, were excluded from this study. All patients were regularly followed up every 3–6 months or in case of necessity (disease flare and/or safety issues).

### Ethical statement

The present study adheres to the principles outlined in the Declaration of Helsinki and received approval from the local Ethics Committee of the University of Siena (Reference no. 14951). All patients or their legal guardians have provided written informed consent.

### Aims and endpoints

The primary aim of the study was to identify potential ocular variables associated with the development of ME during the ocular disease course. Secondary aims were established as (i) the identification of demographic and clinical characteristics associated with the occurrence of ME, (ii) predictive factors of ME, (iii) its functional impact, and (iv) the influence of diagnostic delay and ocular relapses in the onset of ME. The primary endpoint was analyzed for potential statistical differences between patients with and without ME according to the following variables: anatomical classification, retinal vasculitis, and the presence of other non-steroid-induced ocular structural complications defined as any complication different from steroid-induced cataract and steroid-induced ocular hypertension. Secondary aims were examined by (i) detection of any statistical significant difference between patients with and without ME according to gender, ethnicity, associated systemic disease, positivity of HLA-B*27 and/or HLA-B*51, (ii) binary logistic regression used to detect predictive factors of the development of ME, (iii) any difference in BCVA between patients with and without ME, and (iv) and potential differences in mean diagnostic delay and mean uveitis relapses between groups. The diagnosis of ME was based on clinical, OCT, and/or angiographic findings (increased retinal thickness >300 μm on OCT), the presence of cystoid spaces, and subretinal fluid accumulation visible on OCT or fluorescein angiography. The diagnosis of retinal vasculitis was based on angiographic findings.

### Statistical analysis

Data was analyzed using IBM SPSS Statistics for Windows (IBM Corp., Armonk, NY, US). The Shapiro–Wilk test was used to assess the normality distribution of continuous variables. Mean and standard deviation (SD) or median and interquartile range (IQR) as necessary were employed for quantitative variables, while qualitative variables are reported as absolute frequencies and percentages. Cross-tables were analyzed by Pearson’s Chi-square test, and contingency tables with dimensions greater than 2×2 were also followed by *post-hoc* analysis with adjusted residuals, while differences in mean/median between groups were tested with the Mann–Whitney U test. The odds ratio (OR) with a 95% confidence interval (CI) was calculated in cases of significant results in the Chi-square test, to quantify the odds of ME between two different groups. Potential predictors of ME development were identified by binary logistic regression with the backward stepwise method. The significance threshold was established at a *p*-value of 0.05, and all tests were two-sided.

## Results

**Figure 1:**
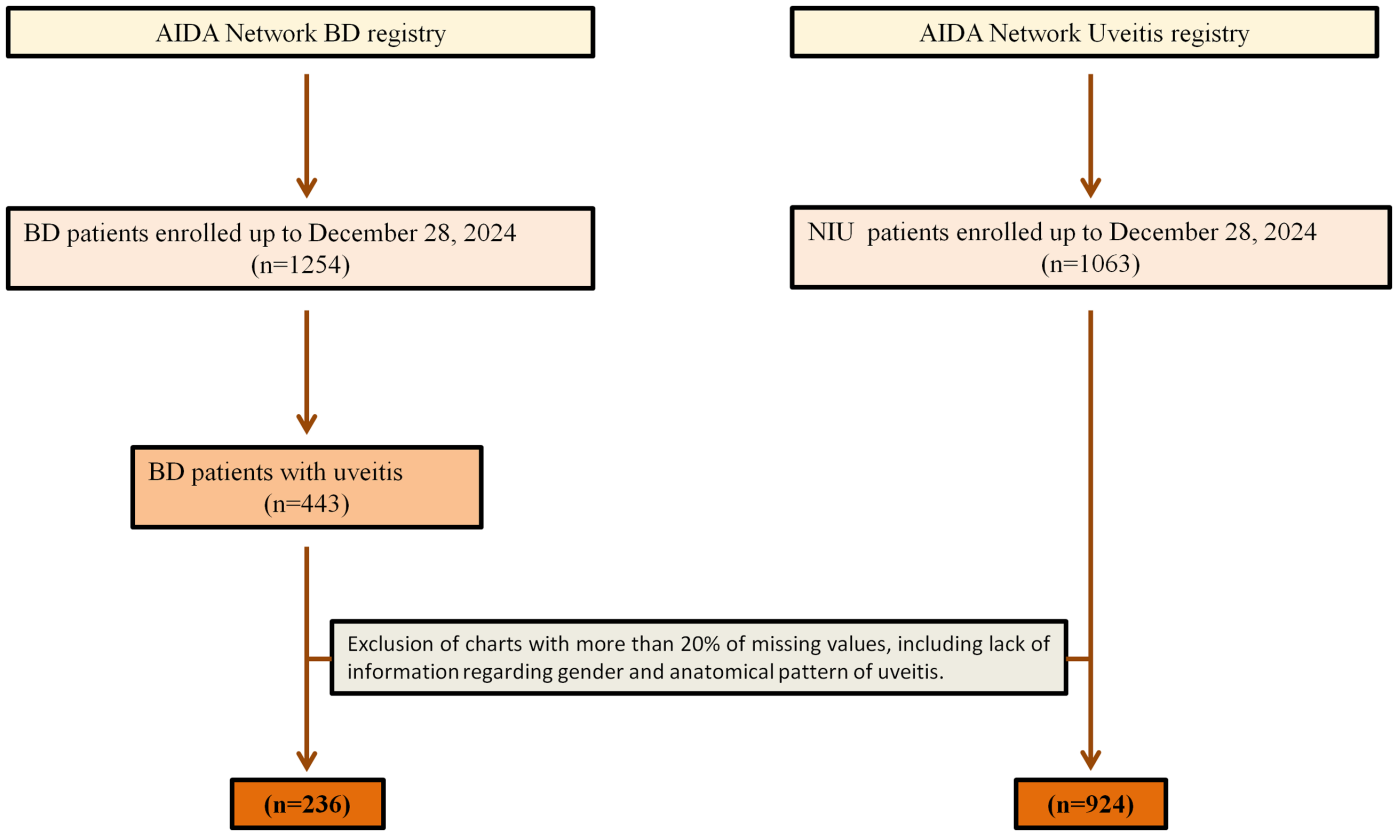
Chart showing the selection process for the patients enrolled. AIDA, Autoinflammatory Disease Alliance; NIU, non-infectious uveitis.

**Figure 2:**
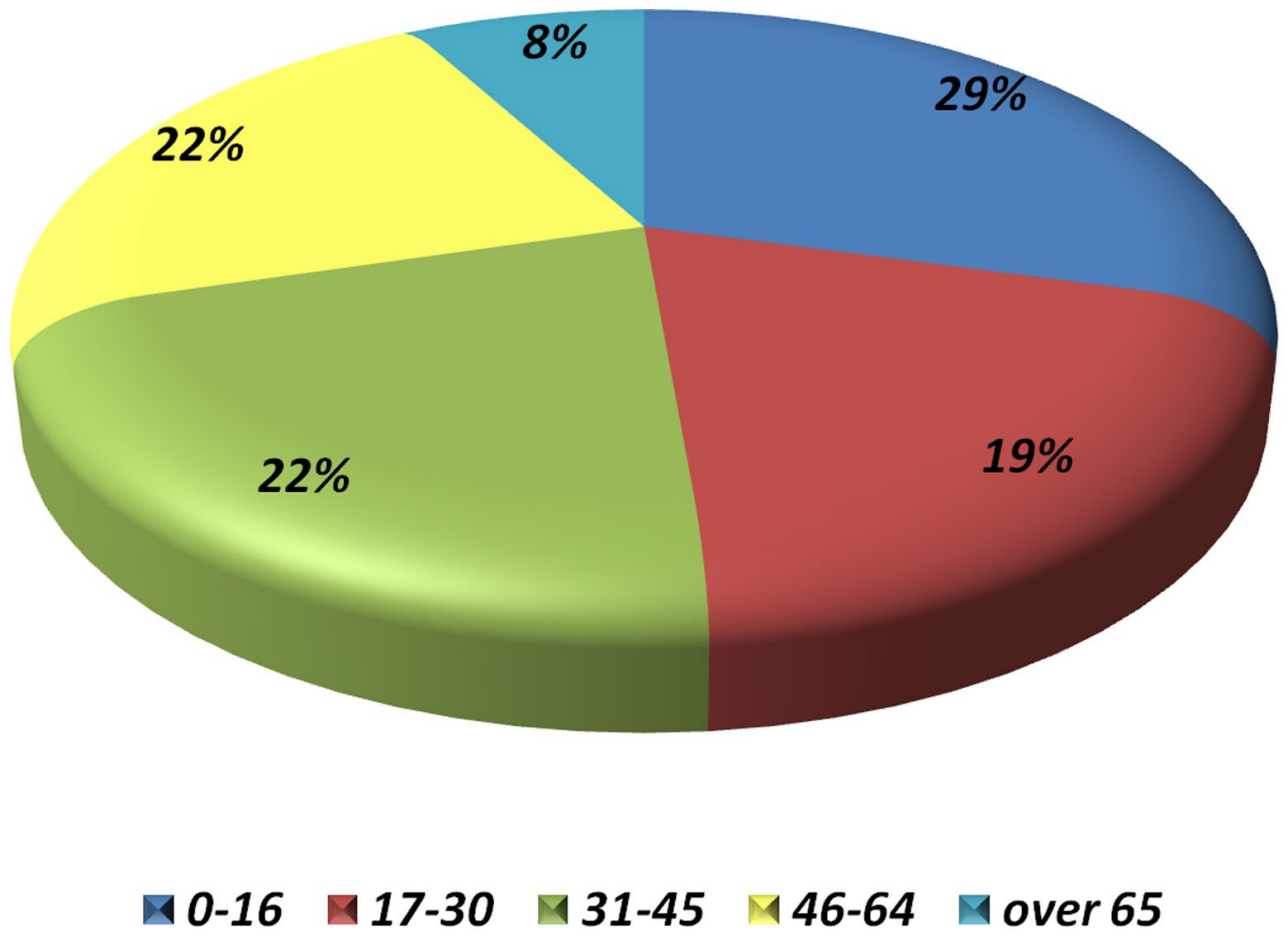
Dsitrubtion of age at onset for pre-established age groups, expressed in years.

**Figure 3:**
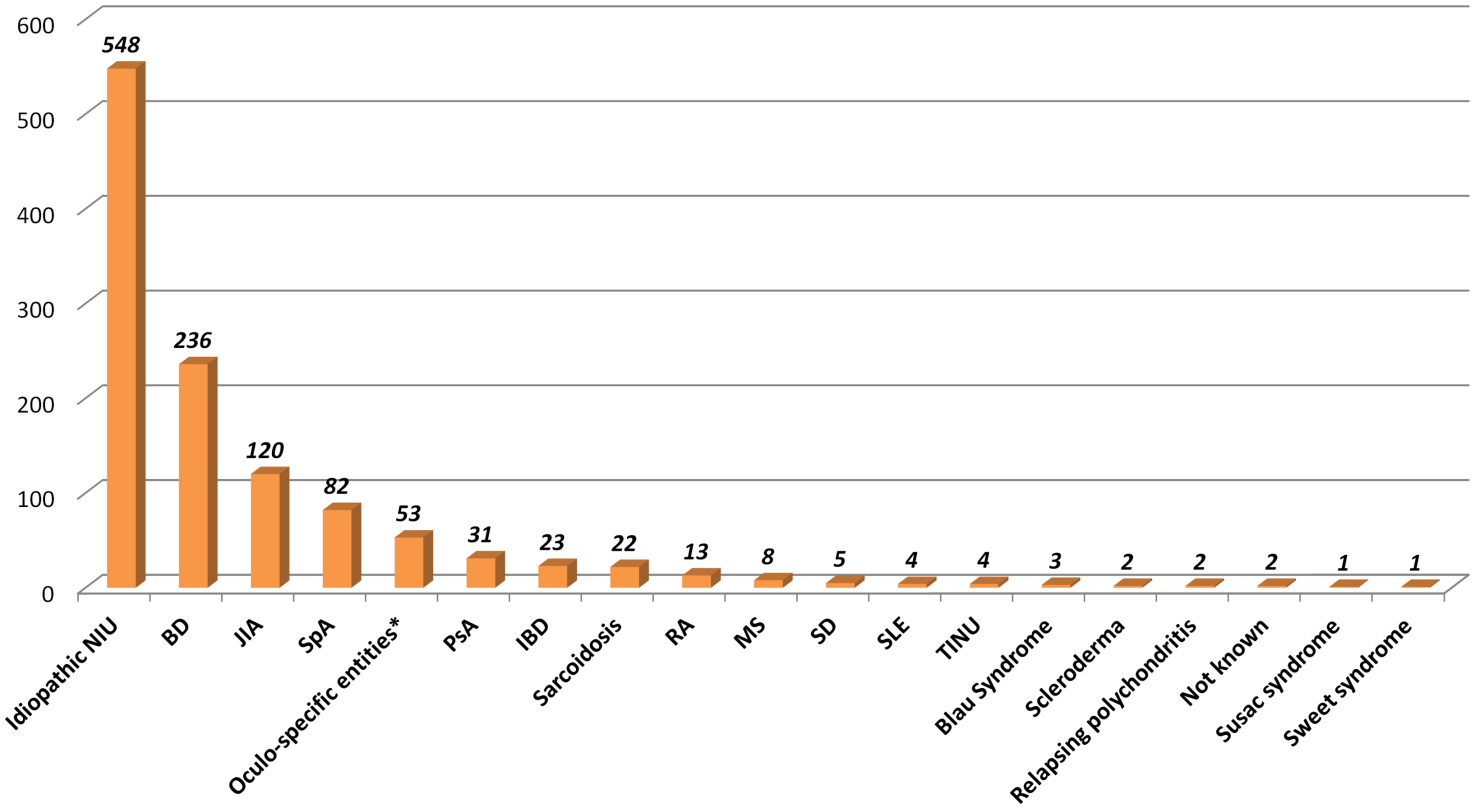
Associated systemic diseases and oculo-specific entities. IBD, inflammatory bowel disease; JIA, juvenile idiopathic arthritis; NIU, non-infectious uveitis; SpA, Spondyloarthritis; TINU, Tubulointerstitial nephritis and Uveitis; SLE, systemic lupus erythematosus; VKH, Vogt-Koyanagi Harada. *Human Leukocyte Antigen-B*27-associated uveitis (*n* = 6), Birdshot retinochoroiditis (*n* = 6), Punctate Inner Choroidopathy (*n* = 4), Serpiginous choroiditis (*n* = 2), Sympathetic ophthalmia (*n* = 1), Idiopathic retinal vasculitis, aneurysms and neuroretinitis (IRVAN) (*n* = 1), not specified (*n* = 6).

We enrolled 1,160 NIU patients representing a total of 1857 eyes, with a female-to-male ratio of 1.32. The ocular involvement was bilateral in 697 cases and unilateral in 463 patients. The ethnic origin was composed mainly of Caucasian (*n* = 770) and Hispanic (*n* = 188) patients, followed by Arab (*n* = 94), African (*n* = 10), Asian (*n* = 10) patients, other (*n* = 4), and unspecified (*n* = 84). Figure 1 displays the cohort selection process and patients excluded from the study for any specific reason. The mean ± SD [median (IQR)] age at the onset of the entire cohort was 33.29 ± 21.39 [32.10 (36.20)] years. The distribution of age at onset according to pre-established age groups is shown in Figure 2. Table 1 lists demographic and clinical features. Regarding the associated systemic immune-mediated diseases, 525 (45.26%) patients were considered to have idiopathic NIU, followed by BD (*n* = 236, 20.34%), juvenile idiopathic arthritis (*n* = 120, 10.34%), and seronegative spondyloarthritis (*n* = 113, 9.7%). Figure 3 details all the associated immune-mediated systemic diseases as well as oculo-specific entities. Overall, ME was observed in 148 (12.76%) patients (211 eyes) throughout the disease course, of whom 24 had concurrent macular epiretinal membranes (MER). Macular epiretinal membranes were registered in 45 patients (3.9%) in the entire cohort. The 24 patients with concurrent ME and MER were excluded from the final statistical analysis to avoid a possible non-uveitic origin of ME. ME was found to be significantly more frequent among patients with non-anterior NIU (*p* < 0.0001). The OR for non-anterior NIU was 4.65, CI (2.96–7.28). Patients with anterior uveitis developed ME in 4.3% of cases, while the subgroups with pars planitis/intermediate uveitis, posterior uveitis, and panuveitis experienced ME in 15.3, 16.6 and 18.6%, respectively. ME was significantly more prevalent in patients with retinal vasculitis (OR 2.89, CI 1.86–4.46, *p* < 0.0001) and in patients presenting with other non-steroid-induced structural complications (OR 1.95, CI 1.34–2.85, *p* = 0.0005). Patients with an associated systemic immune-mediated disease were significantly less likely to experience ME during the NIU course (OR 0.68, CI 0.46–0.99, *p* = 0.042). NIU cases not associated with an immune-mediated systemic disease received fewer immunosuppressive treatments than those associated with a systemic immune-mediated disease (*p* < 0.0001). No significant differences were detected in terms of gender (*p* = 0.635), ethnicity (*p* = 0.193), HLA-B*27 or HLA-B*51 positivity (*p* = 0.323) between NIU patients without and with ME. Table 2 provides details regarding the absolute frequency of ME across several variables, along with their respective level of significance. Four variables were associated with the development of ME during NIU disease course: the non –anterior anatomical pattern (OR = 4.01, CI 2.41–6.67, *p* < 0.0001), the presence of retinal vasculitis (OR = 1.69, CI 1.06–2.67, *p* = 0.028), complications other than ME (OR = 1.51, CI 1.01–2.56, *p* = 0.044), and treatment with immunosuppressive agents during follow-up (OR 1.686, CI 1.131–2.514, *p* = 0.10). On the other hand, the diagnosis of BD-related uveitis is associated with a decreased likelihood of ME development compared to idiopathic NIU (OR 0.545, CI 0.32–0.92, *p* = 0.024). Mean ± SD BCVA was significantly lower in eyes with ME (0.82 ± 0.30) compared to eyes without ME (0.71 ± 0.33) (*p* < 0.0001). The median (IQR) diagnostic delay and the median (IQR) overall number of uveitis relapses experienced during the disease course were not significantly different between patients with and without ME (*p* = 0.472, *p* = 0.321, respectively).

**Table 1:** Demographic and clinical characteristics for each subgroup were examined as well as for the entire cohort.

	NIU	BD-uveitis	Overall cohort
*n* of patients	924	236	1,160
Mean ± SD age at onset (years)	33.26 ± 22.52	33.50 ± 13.30	33.29 ± 21.39
Median (IQR) diagnostic delay (years)	0 (0.1)	0.5 (2)	0 (0.2)
Female/Male	581/343	80/156	661/499
Laterality	Monolateral (*n* = 380) Bilateral (*n* = 544)	Monolateral (*n* = 83) Bilateral (*n* = 153)	Monolateral (*n* = 463) Bilateral (*n* = 697)
Anatomical Pattern* (*n* of eyes, %)	AU (*n* = 545, 59.0%) IU (*n* = 78, 8.4%) PU (*n* = 80, 8.7%) PaU (*n* = 221, 23.9%)	AU (*n* = 53, 22.4%) IU (*n* = 20, 8.5%) PU (*n* = 83, 35.2%) PaU (*n* = 80, 33.9%)	AU (*n* = 598, 51.6%) IU (*n* = 98, 8.5%) PU (*n* = 163, 14.0%) PaU (*n* = 301, 25.9%)

AU, anterior uveitis; BD, Behçet’s disease; IU, intermediate uveitis; NIU, non-infectious uveitis not associated with BD; PanU, panuveitis; PU, posterior uveitis; SD, standard deviation.

**Table 2:** Frequency of macular edema according to different variables taken into consideration.

Variable	ME+	ME−	*p*-value
Anterior uveitis	26	572	<0.0001
Non anterior uveitis	98	464	
Retinal vasculitis	35	124	<0.0001
No retinal vasculitis	89	912	
Other ocular complications	74	447	0.0005
Absence of other ocular complications	50	589	
Males	56	443	0.635
Females	68	589	
HLA-B*27 or HLA-B*51 positivity	11	123	0.323
HLA-B*27 or HLA-B*51 negativity	113	913	

HLA, human leukocyte antigen; ME, macular edema.

## Discussion

Macular edema (ME) plays a major role in visual impairment in NIU patients, making it a pivotal factor in disease management. It is also an underestimated public health issue with a major impact on the quality of life and working ability (1). In our cohort, approximately 13% of all patients developed ME over the course of NIU disease, with the majority of them presenting a non-anterior pattern. Indeed, from an anatomical perspective, the site of inflammation less frequently associated with the development of ME was the anterior segment. More specifically, only 4.3% of patients with anterior uveitis developed ME during their disease course, whereas patients with intermediate uveitis/pars planitis, posterior uveitis, and panuveitis experienced concurrent ME in 15.3, 16.6 and 18.6% of cases, respectively. Alongside the lower prevalence, patients with anterior uveitis appear to present a more favorable prognosis. Matas et al. found that the anterior location carries a higher chance of sustained visual recovery. Eyes with anterior uveitis exhibited more readily sustained gains in vision than those with intermediate, posterior, or panuveitis (8). In our cohort, the anatomical pattern of uveitis was also the strongest predictor of ME development in the multivariate analysis, with non-anterior NIU patients displaying a likelihood of roughly 4 times greater than patients with anterior uveitis. Patients with retinal vasculitis were also more likely to develop ME throughout the disease course. This finding aligns with previous data reporting twice the risk (HR 2.2) of ME in NIU with retinal vasculitis compared to eyes without retinal vasculitis (9). In this regard, from a pathogenetic point of view, vascular damage results in loss of vessel wall integrity and subsequently leads to the retinal extracellular space with cystoid ME development (10). We found a significantly higher rate of ME in idiopathic NIU and NIU related to oculo-specific entities compared to NIU associated with systemic immune-mediated diseases. This finding was corroborated in the regression analysis, where patients with BD-related uveitis were found to be less likely to develop ME during follow-up analysis. A possible explanation could be that patients affected with systemic immune-mediated diseases significantly received more immunosuppressants than patients with idiopathic NIU, potentially altering disease course. Similarly, the presence of non-steroid ocular induced complications and treatment with immunosuppressive agents were associated with a significantly higher occurrence rate of ME, possibly reflecting a suboptimal treatment strategy and/or a more severe ocular disease. The association of a higher prevalence of ME in cases treated with immunosuppressive agents likely indicates a confounding by indication, as more severe patients are more prone to develop ME and to receive aggressive immunosuppressive treatment. Therefore, the higher rate of ME among patients on immunosuppressants does not imply a causal relationship but rather underscores the severity of the underlying disease. As a consequence, immunosuppressive treatment remains protective; however, its benefits could be masked in observational data due to this confounding. Neither gender, ethnicity, nor HLA typing significantly impacted the occurrence rate of ME, highlighting a minor influence of these factors on the development of ME throughout the disease course. Taken together, these findings suggest that ME is significantly more prone to complicate the disease course in NIU patients with posterior segment involvement, supporting the hypothesis of the local pathogenesis theory, while extra-ocular factors appear to be less relevant. The myriad of inflammatory mediators modify the equilibrium of the retinal milieu, leading to a loss of integrity of the inner and/or outer blood retinal barrier, followed by fluid accumulation. In addition, measurements of cytokine and chemokine levels in aqueous humor in uveitis patients with and without ME have revealed high levels of IL-6 (11, 12). This finding could also open new avenues from a therapeutic perspective, considering the superiority of IL-6 blockers in determining a complete response to uveitic ME compared to anti-TNF-*α* monoclonal antibodies (13). Regarding visual acuity, as expected, ME determined a significant impact on BCVA measured at the last follow-up visit. In a large cross-sectional study on the impact of uveitic ME on visual acuity, 44% of patients with ME had a visual acuity of 20/60 or less in at least one eye (14). ME is undoubtedly a major determinant of poor visual acuity, accounting for 40% of cases with visual impairment in uveitis patients (1). There is a growing recognition that considers ME as part of the active disease process during uveitis rather than merely a structural complication. The elevated cytokine and chemokine levels observed in the aqueous humor of uveitis patients with ME imply an inflammatory origin directly tied to uveitis activity (15, 16). In addition, uveitic ME may also reflect the persistence of an underlying ongoing inflammatory process, regardless of the overall number of uveitis relapses. Furthermore, according to our current data, the frequency of ocular relapses does not correlate with an increased occurrence of ME. Therefore, an important consideration would be to view ME as influenced by factors other than inflammatory intraocular attacks, such as chronic or subclinical inflammation. Whether ME is considered part of the active disease process or as a structural complication, its management is pivotal in preventing long-term visual impairment, and a prompt intervention is required to avoid irreversible changes in retinal layers. Specific conditions, such as posterior segment involvement and retinal vasculitis, could serve as drivers to assist physicians in making therapeutic decisions and orienting a step-down approach rather than the more commonly adopted step-up choice. Several study limitations should be mentioned. First, registry-based studies are accompanied by inherent shortcomings such as differences in patient management practices across centers and the lack of standardized follow-up protocols. Secondly, detailed therapeutic data and outcome measures related to treatment response were not retrieved. As a consequence, the impact of each therapeutic agent and its influence as a confounding factor were not assessed. Thirdly, the heterogeneity of the cohort in terms of various underlying diagnoses, while enhancing external validity and reflecting real-life clinical practices, introduces variability in disease pathophysiology, treatment responses, and risk profiles for ME. Another limitation of the present study is the lack of detailed data on OCT imaging and fluorescein angiography, such as RPE abnormalities, site of leakage, and type of retinal vasculitis, which are key markers that could predict ME and/or influence its persistence. Finally, data are mainly collected from reference tertiary referral centers, which examine patients with a more severe disease course, thus generating a potential referral bias and missing a considerable portion of milder cases. This aspect may overestimate the occurrence of ME and/or the strength of association with certain clinical features, limiting the applicability of the findings to populations seen in primary or secondary care settings.

In summary, ME can occur in all NIU subtypes, including anterior uveitis, and complicate the disease course in such patients. However, it is more likely to occur in cases with posterior segment involvement or panuveitis and in patients with retinal vasculitis. Therefore, this subgroup of patients should be carefully monitored on a tight follow-up schedule in order to initiate effective treatment, aiming to prevent the development of ME and subsequent progressive visual loss.

## Data Availability

Raw data supporting the conclusions of this article will be made available by the authors, upon reasonable request.

## References

[1] Daruich A Matet A Moulin A Kowalczuk L Nicolas M Sellam A. Mechanisms of macular edema: beyond the surface. Prog Retin Eye Res. (2018) 63:20–68. doi: 10.1016/j.preteyeres.2017.10.006, PMID: 29126927

[2] Teper SJ. Update on the Management of Uveitic Macular Edema. J Clin Med. (2021) 10:4133. doi: 10.3390/jcm10184133, PMID: 34575244 PMC8470573

[3] Rothova A. Medical treatment of cystoid macular edema. Ocul Immunol Inflamm. (2002) 10:239–46. doi: 10.1076/ocii.10.4.239.15589, PMID: 12854032

[4] Tomkins-Netzer O Lightman SL Burke AE Sugar EA Lim LL Jaffe GJ. Seven-year outcomes of uveitic macular edema: the multicenter uveitis steroid treatment trial and follow-up study results. Ophthalmology. (2021) 128:719–28. doi: 10.1016/j.ophtha.2020.08.035, PMID: 32918964 PMC7943640

[5] Vitale A Della Casa F Ragab G Almaghlouth IA Lopalco G Pereira RM. Development and implementation of the AIDA international registry for patients with Behçet's disease. Intern Emerg Med. (2022) 17:1977–86. doi: 10.1007/s11739-022-03038-1, PMID: 35831701 PMC9522756

[6] Casa FD Vitale A Guerriero S Sota J Cimaz R Ragab G. Development and implementation of the AIDA international registry for patients with non-infectious uveitis. Ophthalmol Ther. (2022) 11:899–911. doi: 10.1007/s40123-022-00459-1, PMID: 35099782 PMC8927492

[7] Jabs DA Nussenblatt RB Rosenbaum JT Standardization of Uveitis Nomenclature (SUN) Working Group. Standardization of uveitis nomenclature for reporting clinical data. Results of the first international workshop. Am J Ophthalmol. (2005) 140:509–16. doi: 10.1016/j.ajo.2005.03.057 16196117 PMC8935739

[8] Matas J Llorenç V Fonollosa A Esquinas C Diaz-Valle D Berasategui B. Predictors for functional and anatomic outcomes in macular edema secondary to non-infectious uveitis. PLoS One. (2019) 14:e0210799. doi: 10.1371/journal.pone.0210799, PMID: 30677041 PMC6345485

[9] Sharief L Lightman S Blum-Hareuveni T Bar A Tomkins-Netzer O. Clinical outcome of retinal Vasculitis and predictors for prognosis of ischemic retinal Vasculitis. Am J Ophthalmol. (2017) 177:206–12. doi: 10.1016/j.ajo.2017.02.028, PMID: 28263735

[10] El-Asrar AM Herbort CP Tabbara KF. A clinical approach to the diagnosis of retinal vasculitis. Int Ophthalmol. (2010) 30:149–73. doi: 10.1007/s10792-009-9301-3, PMID: 19190857

[11] Rothova A. Inflammatory cystoid macular edema. Curr Opin Ophthalmol. (2007) 18:487–92. doi: 10.1097/ICU.0b013e3282f03d2e, PMID: 18163001

[12] Yang JY Goldberg D Sobrin L. Interleukin-6 and macular edema: a review of outcomes with inhibition. Int J Mol Sci. (2023) 24:4676. doi: 10.3390/ijms24054676, PMID: 36902105 PMC10003386

[13] Leclercq M Andrillon A Maalouf G Sève P Bielefeld P Gueudry J. Anti-tumor necrosis factor α versus tocilizumab in the treatment of refractory Uveitic macular edema: a multicenter study from the French uveitis Network. Ophthalmology. (2022) 129:520–9. doi: 10.1016/j.ophtha.2021.11.013, PMID: 34793830

[14] Lardenoye CW van Kooij B Rothova A. Impact of macular edema on visual acuity in uveitis. Ophthalmology. (2006) 113:1446–9. doi: 10.1016/j.ophtha.2006.03.027 16877081

[15] Fardeau C Champion E Massamba N LeHoang P. Uveitic macular edema. Eye (Lond). (2016) 30:1277–92. doi: 10.1038/eye.2016.115, PMID: 27256304 PMC5129852

[16] de Smet MD. Insights into the physiopathology of inflammatory macular edema. Dev Ophthalmol. (2017) 58:168–77. doi: 10.1159/000455279, PMID: 28351051

